# Sleep quality, sensory processing abilities and work performance for adults with attention deficit hyperactive disorder

**DOI:** 10.1192/j.eurpsy.2021.1475

**Published:** 2021-08-13

**Authors:** N. Grinblat, S. Rosenblum

**Affiliations:** Occupational Tharepy, University of Haifa, Haifa, Israel

## Abstract

**Introduction:**

Poor sleep quality has been reported among adults with attention-deficit hyperactive disorder (ADHD) and has been associated with reduced sensory-processing abilities and low work performance. However, the relationships among sleep quality, sensory processing and the insufficient work performance of adults with ADHD is still unclear.

**Objectives:**

Following the World Health Organization’s international classification of functioning, disability and health concepts, this study compares sleep quality and sensory processing (body functions) and work performance (participation) of adults with ADHD to those of controls, and examines the relationships among those components in adults with ADHD.

**Methods:**

Participants were 69 adults with ADHD and 52 age- and gender-matched controls. All completed a sociodemographic questionnaire, the Mini Sleep Questionnaire, the Adult/Adolescent Sensory Profile and the Occupational Questionnaire.

**Results:**

Compared to controls, the adults with ADHD showed significantly poorer body functions (sleep quality and sensory processing patterns) and lower work performance. Significant correlations were found between sleep quality and sensory-processing abilities and between sleep quality and work performance among adults with ADHD. Regression analysis revealed that for adults with ADHD, sleep quality accounted for 22.0%, and sensory sensitivity accounted for 10.9%, of the variance of their work performance.

**Conclusions:**

Sleep quality, together with sensory processing patterns, has a significant influence on work performance of adults with ADHD. Because work is a vital occupation for adults, these body functions need to be considered in clinical settings, and further research on this topic is required for better understanding of the phenomena.

**Keyword:**

ADHD add Sleep quality add work performance add sensory processing

